# First and Second Language Reading Difficulty Among Chinese–English Bilingual Children: The Prevalence and Influences From Demographic Characteristics

**DOI:** 10.3389/fpsyg.2019.02544

**Published:** 2019-11-15

**Authors:** Yue Gao, Lifen Zheng, Xin Liu, Emily S. Nichols, Manli Zhang, Linlin Shang, Guosheng Ding, Xiangzhi Meng, Li Liu

**Affiliations:** ^1^State Key Laboratory of Cognitive Neuroscience and Learning and IDG/McGovern Institute for Brain Research, Beijing Normal University, Beijing, China; ^2^Max Planck Institute for Psycholinguistics, Nijmegen, Netherlands; ^3^Department of Physics and Astronomy, University of Western Ontario, London, ON, Canada; ^4^Maastricht Brain Imaging Center, Department of Cognitive Neuroscience, Faculty of Psychology and Neuroscience, Maastricht University, Maastricht, Netherlands; ^5^School of Psychological and Cognitive Sciences, Beijing Key Laboratory of Behavioral and Mental Health, Peking University, Beijing, China; ^6^PekingU-PolyU Center for Child Development and Learning, Beijing, China

**Keywords:** reading difficulty, Chinese–English bilinguals, sex differences, urban–rural gap, first language, second language

## Abstract

Learning to read a second language (L2) can pose a great challenge for children who have already been struggling to read in their first language (L1). Moreover, it is not clear whether, to what extent, and under what circumstances L1 reading difficulty increases the risk of L2 reading difficulty. This study investigated Chinese (L1) and English (L2) reading skills in a large representative sample of 1,824 Chinese–English bilingual children in Grades 4 and 5 from both urban and rural schools in Beijing. We examined the prevalence of reading difficulty in Chinese only (poor Chinese readers, PC), English only (poor English readers, PE), and both Chinese and English (poor bilingual readers, PB) and calculated the co-occurrence, that is, the chances of becoming a poor reader in English given that the child was already a poor reader in Chinese. We then conducted a multinomial logistic regression analysis and compared the prevalence of PC, PE, and PB between children in Grade 4 versus Grade 5, in urban versus rural areas, and in boys versus girls. Results showed that compared to girls, boys demonstrated significantly higher risk of PC, PE, and PB. Meanwhile, compared to the 5th graders, the 4th graders demonstrated significantly higher risk of PC and PB. In addition, children enrolled in the urban schools were more likely to become better second language readers, thus leading to a concerning rural–urban gap in the prevalence of L2 reading difficulty. Finally, among these Chinese–English bilingual children, regardless of sex and school location, poor reading skill in Chinese significantly increased the risk of also being a poor English reader, with a considerable and stable co-occurrence of approximately 36%. In sum, this study suggests that despite striking differences between alphabetic and logographic writing systems, L1 reading difficulty still significantly increases the risk of L2 reading difficulty. This indicates the shared meta-linguistic skills in reading different writing systems and the importance of understanding the universality and the interdependent relationship of reading between different writing systems. Furthermore, the male disadvantage (in both L1 and L2) and the urban–rural gap (in L2) found in the prevalence of reading difficulty calls for special attention to disadvantaged populations in educational practice.

## Introduction

Reading is a foundational and crucial cognitive skill for children to become participating and contributing members in the global society. However, for some children, despite having normal intelligence and adequate education, reading is a struggle rather than an enjoyment (Stevenson et al., 1982; Chan et al., 2007). An additional challenge is that children may have to learn to read a second language (L2) at the same time due to political, social, educational, or personal reasons (Gunderson et al., 2011), regardless of whether or not they are struggling with L1 reading. In light of these difficulties, both the prevalence of reading difficulty in L1 and L2 and how varying levels of L1 reading ability affect L2 reading success become important concerns for parents, educators, and researchers.

For second language learners of English, a substantial number of studies have consistently found associations between poor reading in L2 (English) and poor reading in L1, which thus far have mostly been alphabetic languages as in the case of Spanish (Lindsey et al., 2003), Italian (D’Angiulli et al., 2001), French (Deacon et al., 2009), Dutch (Morfidi et al., 2007), Hebrew (Geva and Siegel, 2000), and Korean (Wang et al., 2006). These results demonstrate an interdependent relationship between poor reading skills in L1 and L2. A number of hypotheses, such as the Linguistic Interdependence Hypothesis (Cummins, 1979, 1981), the Linguistic Coding Differences Hypothesis (LCDH, Sparks, 1995), and the Central Processing Hypothesis (Geva and Siegel, 2000) have all stated that deficits in L1 and L2 reading may share common cognitive bases or linguistic components (Geva and Siegel, 2000). Therefore, children struggling to read in their L1 may also face challenges when learning to read a foreign language as an interdependent result. However, other theories, like the Orthographic Depth Hypothesis (Katz and Frost, 1992) and the Psycholinguistic Grain Size (Ziegler and Goswami, 2005), have posited that poor L2 reading abilities may be due to inadequately meeting the demands of the L2. According to these theories, students learning to read an opaque language as an L2 may face problems as they lack the strategies and training in whole word recognition (Abu-Rabia et al., 2013; Chung et al., 2018). Similarly, pupils learning to read an L2 with a more transparent orthography might also struggle, as they are not familiar with the grapheme–phoneme correspondence rule.

Both sets of theories were shown to be plausible with the discovery of children who were experiencing reading difficulty in either purely English (L2), or in both Chinese (L1) and English (L2), who were learning these two vastly different writing systems in primary schools in Beijing (McBride-Chang et al., 2013) and Hong Kong (Ho and Fong, 2005; Chung and Ho, 2010; McBride-Chang et al., 2013; Tong et al., 2015). In these studies, the existence of poor English only readers provided evidence of individual differences in reading in two writing systems, suggesting that L1 and L2 reading might demand different cognitive skills. In comparison, the cross-language transfer of certain reading-related skills suggested universal linguistic underpinnings for reading in two languages. Results showed that in Beijing, 40% of the poor Chinese (L1) readers were also poor English (L2) readers. This co-occurrence, i.e., the rate of poor Chinese (L1) readers also being poor English (L2) readers was significantly above the baseline level, suggesting that poor L1 reading increased the likelihood of L2 reading problems. However, studies (McBride-Chang et al., 2013; Tong et al., 2015) have shown that reading difficulty co-occurrence among children aged 8 (approximately second graders) and aged 10 years (approximately fifth graders) is different in Hong Kong, with the former being 32% (similar to the co-occurrence in Beijing) and the latter being 57%. This might be due to development: by age 8 years, though they would have had sufficient exposure to English (McBride-Chang et al., 2013), children have started to shift from “learning to read” to “reading to learn.” In the latter stage, children need to use reading as a tool to build vocabulary and knowledge, thus posing greater challenges to those students in higher grades for both L1 and L2. On the other hand, this might also be due to the relatively small sample sizes in these studies (McBride-Chang et al., 2013: Age 8 children: *N* = 147; Tong et al., 2015: Grade 5 children: *N* = 162). Therefore, it is necessary to conduct a large-scale study to examine the prevalence of PC, PE, and PB, and more importantly, the risks for poor L2 reading among poor L1 readers in older children, who have more reading experience in both languages. Understanding how and to what extent L2 reading is affected as L1 reading ability varies is important.

With that being said, factors that put children at risk for reading difficulty, particularly the urban–rural gap, and those related to students’ characteristics have not been well addressed. Several socio-demographic characteristics have been suggested to affect the prevalence of reading difficulty. Among them, sex differences in reading performance have been found and replicated in numerous studies (Shaywitz et al., 1990; Rutter et al., 2004; Stoet and Geary, 2013; Quinn, 2018) and shown to not be due to sampling and measurement procedures (Arnett et al., 2017), ascertainment bias (in which males are more likely to be referred for evaluation than females with equivalent reading problems) (Quinn and Wagner, 2015), nor unequal educational opportunities for females (OECD, 2016b). In addition to sex influences, the effect of grade level is also evident. In a study of reading-related skills in native Chinese speaking children, researchers (Lei et al., 2011) found that one group of children, despite initial early deficits in phonological and morphological awareness, caught up with the peers and acquired adequate subsequent reading ability 3 years later. These results suggested that, as children enter higher grades and receive more training, their language reading ability gradually develops. Higher graders might also acquire more reading strategies and gain more reading experiences.

School location, which reflects the school’s socio-economic status, also influences children’s reading achievement (Xuan et al., 2019). China has experienced unprecedented economic growth in the past few decades, and rather than benefiting the urban and rural residents equally, the growth has widened the existing gap between urban and rural regions (Sicular et al., 2007). The situation makes the contrast in urban–rural schooling in China a very special case and worth more investigation and comparison.

However, effects of sex, school location, and grade level have been mostly reported in L1 reading and to the best of our knowledge, no previous studies have investigated the influences of these demographic characteristics on the prevalence of reading difficulty in English as L2 in native Chinese speakers. Further, the demographic influences on the prevalence of L1 Chinese reading difficulty are also sparse. Despite the observed male disadvantage in the prevalence of L1 Chinese reading difficulty in a few studies (Chan et al., 2007; Song and Wu, 2008; Zuo et al., 2010), compared to the abundant studies conducted in alphabetic languages, very little is known about what factors increase vulnerability for reading difficulty in Chinese. Finally, it is unclear whether the rate of co-occurrence, i.e., the chance of being a poor L2 reader among children who are identified as poor L1 readers, also demonstrates socio-demographic differences. Therefore, studying demographic characteristics, understanding the associations between these characteristics with poor reading, and expanding our understanding from poor reading in L1 to poor reading in L2 can capture more accurately and more fully the influences that shape children’s bilingual reading.

Here, we answer these questions within the framework of Chinese–English bilingual reading. We were interested in examining Chinese (L1) and English (L2) reading abilities, with an individual’s ability defined as knowing “how words are identified and related to spoken language processes” (Perfetti, 1985). English has an alphabetic writing system, and following the alphabetic principle, phonological cues in English words contribute greatly to reading. Therefore, weakness in phonological processing, such as phoneme decoding and grapheme–phoneme conversion, may be at the core of children’s struggles with English (L1) reading (Bradley and Bryant, 1983; Snowling, 2001; Ramus, 2014). In comparison, Chinese characters consist of various radicals arranged two-dimensionally, and the phonemic information conveyed via radicals is relatively irregular or limited. Therefore, the cognitive correlates of Chinese reading difficulty may be multifaceted, with phonological processing, orthographic awareness, and visual analysis all heavily involved (Peng et al., 2017). Both the universal and unique characteristics of Chinese and English reading make them a particularly effective pairing for examining the inter-relationship between poor reading in L1 and L2, and for testing theories proposed under the investigation of alphabetic languages. However, remarkably few studies have sought to identify the contribution of demographic characteristics to the prevalence of Chinese and English reading ability relations, thus the question remains as to whether demographic characteristics affect the relationship between L1 and L2 reading difficulty, and whether children’s different backgrounds influence the prevalence of L1 and L2 reading difficulty.

The overarching goal of this study is to examine the relationship of reading difficulty in L1 and L2 in a large sample of Chinese (L1)–English (L2) bilingual children. First, we provide basic prevalence data of reading difficulty in L1 only, in L2 only, and in both L1 and L2. Second, we build a multinomial logistic regression model to compare different types of struggling readers to normal readers, and examine how grade level, sex, and school setting affects their L1 and L2 reading abilities. Finally, we address how and to what extent L1 reading difficulties significantly increase the prevalence of poor L2 reading.

## Materials and Methods

### Participants

Participants’ demographics are described in Table 1. A total of 1,824 Chinese–English bilingual students from primary schools in Beijing were assessed. For each student, we collected data on sets of variables that included demographic characteristics (age, sex, school location, grades), intelligence tests, and reading-related tests (both a Chinese reading test and an English spelling test). Valid data here refer to a dataset with complete reading-related test data and no more than 1 variable data missing from their demographic information. 1,786 of the participants provided valid data (97.92%). Among the children with valid data, there were 976 boys and 805 girls, with 5 of them lacking sex information. There were 880 4th graders and 906 5th graders; 563 students from rural schools and 1,218 from urban schools, with five children lacking school location information. Among all eight schools included in this study, five are located in the downtown area [Haidian District and Chaoyang District, with GDP per capita of $24,590 and $21,442 (USD), and total GDP ranked 1st and 2nd among all 16 districts of Beijing in 2017], another three are located in rural areas [Changping district and Miyun District, with GDP per capita of $5,884 and $8,816 (USD), and total GDP ranked 8th and 13th among all 16 districts of Beijing in 2017]. Additionally, Haidian and Chaoyang Districts are both equipped with more than two public libraries, whereas there are none in Changping and Miyun Districts (Beijing Social Development Database^1^). All children started to receive formal Chinese and English instruction in Grade 1, at approximately 6 years old. Written informed consent was obtained from each participant and their parents. The institutional review board at Beijing Normal University approved the informed consent procedures.

**TABLE 1:** Demographic characteristics and descriptive statistics for all children.

	**Grade 4**	**Grade 5**
Number of children	880	906
Number of boys	460	516
Number of girls	415	390
Number of urban students	576	642
Number of rural students	304	259
Mean age in months (SD)	118.44 ± 6.11	131.00 ± 9.46
Mean non-verbal IQ in percentile (SD)	70.40 ± 22.84	67.24 ± 24.78
Mean Chinese reading score^∗^	2,486 ± 484	2,857 ± 356
Mean English reading score^#^	10.75 ± 8.34	16.08 ± 8.94
Number and prevalence of PC	(101)11.47%	(45)5.00%
Number and prevalence of PE	(123)13.97%	(131)14.46%
Number and prevalence of PB	(48)5.45%	(34)3.75%

*PC, poor Chinese reader; PE, poor English reader; PB, poor reader in both languages. ^∗^Chinese reading score is measured by the Chinese character recognition test (in spelling format), and the score represents how many characters a child can use to make and write down a word. The full score is 3500. ^#^English reading score is measured by an English word spelling test, and the score represents how many words a child can spell. The full score is 40.*

Based on the information we gathered, in urban primary schools students receive four English classes and four Chinese classes in a week (from Monday to Friday, each class takes 45 min), and this is true for both 4th and 5th graders. Urban school students also have the opportunity to attend English-related activities outside the classroom. Similarly, rural schools also provide four English classes and four Chinese classes for the 4th and 5th graders every week. The course arrangements, in both content and frequency, are relatively similar between rural and urban areas. This is because education in China is state-run, and the Ministry of Education of People’s Republic of China is the agency of the State Council that oversees education throughout the country. They “lay down requirements and create basic documents for teaching and curriculum in elementary education; organize the examination and approval of unified course materials for elementary education; and to develop high-quality education in a comprehensive manner (OECD, 2016a).” With the foundation set, policies and strategies designed by the Ministry of Education are implemented by local departments of education under its direct management. For example, to meet the basic requirements for setting up English courses in the primary schools, schools follow the principle of short courses at high frequency and ensure at least three teaching activities per week. Finally, the participants come from Han ethnicity families and schools, indicating that their home language, school language, and social language are all Chinese.

### Behavioral Measures

Raven’s standard progressive matrices: This test is used to assess children’s non-verbal IQ by measuring their general non-verbal reasoning ability. Scoring procedures were based on the Chinese normative data (Zhang and Wang, 1985).

Chinese character recognition test (in writing format): This test (Wang and Tao, 1996) consists of 10 groups of Chinese characters at increasing levels of difficulty. Participants were given 40 min to write down a compound word using each provided character. As there are numerous homophones in Chinese, compound word spelling compared to character reading aloud can better assess whether a child can access character meaning. Additionally, this paper–pencil test can be administered in group and was therefore suitable for large-scale assessment. This standardized test has been widely used to screen Chinese children with reading difficulty (Liu et al., 2012, 2013; McBride-Chang et al., 2013; Peng et al., 2017). The test–retest reliability of this test is 0.970 for Grade 4 and 0.984 for Grade 5.

English word spelling test: We used an English word spelling test to screen poor English readers in Chinese–English bilingual children, as has been previously done (You et al., 2011; Li et al., 2018). Forty English words chosen from primary school English textbooks for Chinese children were included, with half high-frequency words and half low-frequency words. Each word was read aloud twice and the participants were asked to write down the word on the answer sheet. This test can be administered in a group and is suitable for large-sample studies. The test–retest reliability of this test is 0.96. Moreover, to identify the capacity of the spelling test instrument, we used the Word Identification test (Woodcock, 1987) as the screening criteria in a subsample of 94 students and identified students with normal English reading ability as well as those with deficient English reading ability. We then compared the classifying results based on word identification test and classifying results based on the English spelling test, and calculated the sensitivity (93%) and the specificity (83.7%) of the English spelling test. The sensitivity and specificity were calculated based on signal detection theory [sensitivity = True Positive/(True Positive + False Negative), specificity = True Negative/(True Negative + False Positive)] (Green and Swets, 1966). This cross validation suggested that the English word spelling test is a sensitive and valid test to screen poor English readers in Chinese–English bilingual children.

### Criteria for Screening Poor Readers

With parental consent obtained, children’s Chinese and English reading ability was assessed by trained psychology majors in the children’s classroom. The time required for the Chinese reading test varied from 20 to 40 min. The English spelling test took up to 10 min. Both tests were administered with Chinese instructions to ensure that children fully understood the requirements. All administrators passed the College English Test-Band 6 (CET-6), which is a national English standardized test evaluating the English proficiency of undergraduates and postgraduates in People’s Republic of China. The order of the two tests was randomized. At the end of the testing, any questions that participants had were answered. A fairly liberal, but not unusual, criteria was used for screening poor readers (e.g., Francis et al., 2005; McBride-Chang et al., 2013; Tong et al., 2015): first, the percentile in the Raven’s test score had to be above the 40th to ensure a normal IQ; second, for poor Chinese readers (PC, *N* = 146), their performance on the Chinese character recognition test had to fall at or below 25th percentile, whereas their English spelling score had to be above this level. Similarly, to define poor English readers (PE, *N* = 254), their performance in the English spelling test needed to be at or below 25th percentile while their Chinese test score needed to be above this level. Finally, poor bilingual readers (PB, *N* = 82) refer to those whose English and Chinese scores were both falling at or below 25th percentile. Normal readers refer to the students whose Chinese and English reading performance were both above the 25th percentile (NR, *N* = 1,299).

### Data Analysis

First, we computed the prevalence of different types of poor reading (PC, PE, and PB) among Chinese–English bilingual children, for boys versus girls, for urban versus rural, and for Grade 4 versus Grade 5. Second, we applied a multinomial logistic regression, a model that allows for more than two categories of the outcome variables to be predicted, to investigate the association between reading difficulty in L1 and L2 and demographic characteristics. Reading group membership (PC versus NR, PE versus NR, PB versus NR, PC versus PE, PC versus PB, PB versus PE) was used as the criterion variable with demographic characteristics (sex, school location, grades) entered as predictors. Third, we referred to the formula developed by McBride-Chang et al. (2013) to describe the chances of a poor L1 reader concurrently manifesting difficulty in L2 reading (here referred to as co-occurrence): number of poor bilingual readers/(number of poor bilingual readers + number of poor Chinese readers) ^∗^ 100%. The percentage represents the portion of readers who manifested reading difficulty in L1 and L2, among the population with L1 reading difficulty. For the baseline level of L2 reading difficulty, we referred to a second formula (McBride-Chang et al., 2013): number of poor English readers/(number of poor English readers + number of normal readers). The baseline rate represents the percentage of poor L2 only readers among the students without L1 reading difficulty. Based on this, the 2 ^∗^ 2 contingency table was set to compare the frequencies and the co-occurrence was compared with the baseline level of L2 reading difficulty via a non-parametric test (χ^2^) to examine whether L1 reading difficulty would generate a significantly higher occurrence of L2 reading difficulty in boys or girls, in urban or rural areas, and in Grade 4 or Grade 5, respectively.

## Results

**FIGURE 1:**
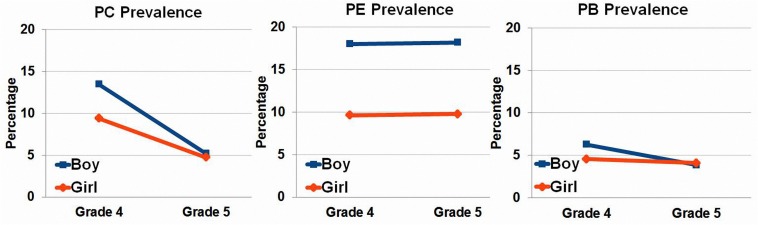
PC, PE, and PB prevalence of boys versus girls, illustrated in Grade 4 and Grade 5 separately. PC, poor Chinese reading; PE, poor English reading; PB, poor bilingual reading.

**FIGURE 2:**
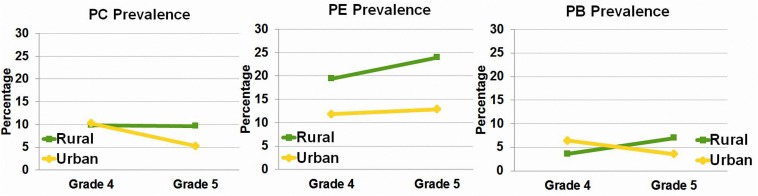
PC, PE, and PB prevalence of rural schools versus urban schools, illustrated in Grade 4 and Grade 5 separately. PC, poor Chinese reading; PE, poor English reading; PB, poor bilingual reading.

Table 1 and Figures 1, 2 present the basic prevalence data for different types of reading difficulty in this sample. The prevalence of PE and PB kept stable in Grades 4 and 5. In contrast, there was a drop in the prevalence of PC in Grade 5 compared to Grade 4. To obtain an overall picture of reading difficulty in Beijing, we collapsed data from the fourth and fifth graders. Results showed that the PB prevalence (4.60%) is lower than the PE (14.22%) and PC (8.20%) prevalence across the whole sample.

Next, we examined the gender, grade, and school location effect on the prevalence of different types of reading difficulty (Table 2). The −2 log likelihood (138.362) and Chi-squared statistics (χ^2^ = 120.0.53, *p* < 0.001) showed that these three predictor variables provided a significant fit to the model. These variables significantly distinguished between the PC and NR groups, between the PE and NR groups, and between the PB and NR groups. In differentiating the PC group from NR group, a one-unit increase in the sex (boys) increased the odds of being in the PC group rather than the NR group by 1/0.521 = 1.919; a one-unit increase in grade (Grade 4) increased the odds of being in the PC group rather than the NR group by 1/0.416 = 2.404. In differentiating the PE group from the NR group, a one-unit increase in sex (boys) increased the odds of being in the PE group rather than the NR group by 1/0.444 = 2.252; a one-unit increase in school location (rural) increased the odds of being in the PE group rather than the NR group by 2.173. In differentiating the PB group from the NR group, a one-unit increase in sex (boys) increased the odds of being in the PB group rather than the NR group by 1/0.442 = 2.262; a one-unit increase in the grade (Grade 4) increased the odds of being in the PB group rather than the NR group by 1/0.588 = 1.701. In differentiating among PC, PB, and PE groups, results consistently showed that the 4th graders were at higher risk than the 5th graders (2.293 times higher in PE than PC, 1.696 times higher in PE than PB), and students in the rural areas were at higher risk than their urban counterparts (1.748 times higher in PE than PC, 1.624 times higher in PE than PB) in manifesting L2 reading difficulty. In summary, being a boy significantly increased the odds of falling into all the three reading difficulty groups. Compared to being in the 5th grade, being in the 4th grade significantly increased the chances of being identified as a poor reader. Being from a rural community particularly influenced poor English reading.

**TABLE 2:** Multinomial logistic regression.

	**NR versus PB**			**NR versus PC**			**NR versus PE**			**PC versus PE**			**PC versus PB**			**PB versus PE**		
	***B*(*SE*)**	**Exp(*B*)**	**95% CI**	***B*(*SE*)**	**Exp(*B*)**	**95% CI**	***B*(*SE*)**	**Exp(*B*)**	**95% CI**	***B*(*SE*)**	**Exp(*B*)**	**95% CI**	***B*(*SE*)**	**Exp(*B*)**	**95% CI**	***B*(*SE*)**	**Exp(*B*)**	**95% CI**
Grade	**−0.531^∗∗^ (0.199)**	0.588	0.398 0.868	**−0.876^∗∗∗^ (0.169)**	0.416	0.299 0.580	**−**0.003 (0.132)	0.997	0.770 1.291	**0.873^∗∗∗^ (0.195)**	2.393	1.633 3.508	0.345 (0.246)	1.411	0.872 2.284	**0.528^∗^ (0.221)**	1.696	1.099 2.617
Sex	**−0.815^∗∗∗^ (0.206)**	0.442	0.296 0.662	**−0.653^∗∗∗^ (0.165)**	0.521	0.377 0.719	**−0.812^∗∗∗^ (0.136)**	0.444	0.340 0.580	**−**0.159 (0.196)	0.853	0.581 1.252	**−**0.163 (0.248)	0.850	0.522 1.383	0.004 (0.231)	0.987	0.638 1.579
School	0.291 (0.209)	1.338	0.888 2.015	0.218 (0.173)	1.243	0.886 1.745	**0.776^∗∗∗^ (0.135)**	2.173	1.668 2.832	**0.559^∗∗^ (0.196)**	1.748	1.190 2.569	0.073 (0.252)	1.076	0.657 1.763	**0.485^∗^ (0.228)**	1.624	1.038 2.542

*95% CI, 95% confidence intervals for Exp *B*; PC, poor Chinese reader; PE, poor English reader; PB, poor reader in both languages; SE, standard error; Exp(B) refers to the odds ratios. ^∗^*p* < 0.05, ^∗∗^*p* < 0.01, ^∗∗∗^*p* < 0.001. The bolded values refer to significant effects.*

To examine whether and to what extent L1 reading difficulties significantly increase the prevalence of performing poorly in L2 reading, we computed the co-occurrence (the chance of poor Chinese readers also being poor English readers) and the baseline level of L2 reading difficulty in the control group (Table 3). No significant sex, location, or grade differences were observed in co-occurrence levels. Co-occurrence was significantly higher than the baseline level regardless of sex (χ^2^ = 13.78, *p* < 0.001 in boys; χ^2^ = 45.44, *p* < 0.001 in girls), school locations (χ^2^ = 50.70, *p* < 0.001 in urban schools; χ^2^ = 3.29, *p* = 0.081 a marginally significant effect in rural schools), and grades (χ^2^ = 18.34, *p* < 0.001 in Grade 4; χ^2^ = 35.81, *p* < 0.001 in Grade 5).

**TABLE 3:** The co-occurrence of L1 and L2 poor reading and the baseline rate of L2 poor reading in different school locations, sex, and grades.

	**Co-occurrence**	**L2 poor reading**	**χ^2^**
Boys Girls Ratio χ^2^	35.51% 36.67% 0.97:1 0.03	26.78% 12.07% 2.22:1	13.78^∗∗∗^ 45.44^∗∗∗^
Urban Rural Ratio χ^2^	36.81% 34.52% 1.07:1 0.13	16.54% 33.52% 0.49:1	50.70^∗∗∗^ 3.29
Grade 4 Grade 5 Ratio χ^2^	32.21% 42.50% 0.76:1 2.63	20.40% 18.82% 1.08:1	18.34^∗∗∗^ 35.81^∗∗∗^

*Co-occurrence refers to the chances of becoming a poor reader in English given that the child was already a poor reader in Chinese. It was calculated by the formula (McBride-Chang et al., 2013): N of PB/(N of PB + N of PC) ^∗^ 100%. The baseline rate of L2 poor reading was calculated by the formula (McBride-Chang et al., 2013): N of PE/(N of PE + N of NR). ^∗^*p* < 0.05, ^∗∗^*p* < 0.01, ^∗∗∗^*p* < 0.001.*

## Discussion

In a large epidemiological sample of Beijing primary school children in Grade 4 and Grade 5, we investigated the prevalence of reading difficulty in L1 (Chinese) and L2 (English) children, and the influence of grade, sex, and school location on reading difficulty. There were three major findings: (1) The co-occurrence rate was significantly higher than baseline levels regardless of sex, school location, and grades. This indicates that being a poor reader in Chinese (L1) significantly increases the risk of also becoming a poor English reader. (2) In general, girls were better at reading in both Chinese and English, shown by the lower risks of all three types of poor reading (PC, PE, and PB). (3) A rural and lower grade disadvantage was observed particularly in PE.

Our first finding, that being a poor reader in Chinese significantly increases the risk of also being a poor English reader, supports theories arguing that deficits in L1 and L2 reading might share some common bases (Cummins, 1979, 1981) or linguistic components (Geva and Siegel, 2000), and involves cross-linguistic transfer from L1 to L2 (Chung and Ho, 2010). We observed a stable co-occurrence of L1 and L2 reading difficulty in Chinese–English bilingual children. Across the sexes and different school locations, the chances of a poor L1 reader showing L2 reading difficulty at the same time were approximately 36%. This probability is not influenced by sex (boys: 35.51%, girls: 36.67%), school location (urban: 36.81%; rural: 34.52%), or grade (Grade 4, 32.21%; Grade 5: 42.50%). The co-occurrences observed are similar to the 40% probability reported in a relatively small sample (*N* = 291, age 8 years) of Beijing primary school children (McBride-Chang et al., 2013), as well as the 32% co-occurrence rate reported in Hong Kong primary school children (*N* = 147, age 8 years) (McBride-Chang et al., 2013). Combining these results with our current study, the co-occurrence of being poor readers in L1 and in L2 appears to be relatively stable in primary school children who learn to read English in Beijing. However, the co-occurrence we observed is smaller than the 57% co-occurrence rate reported among 5th graders in Hong Kong (Tong et al., 2015). Regarding this 57% rate, researchers reported that the sample came from several schools in which children may have been taught similar learning skills or were exposed to similar teaching methods, perhaps increasing the overlap in reading difficulties for Chinese and English (Tong et al., 2015). Alternatively, the observed difference between the co-occurrence of Beijing students and Hong Kong students may reflect the fact that poor reader status in Chinese and English across Beijing and Hong Kong is somehow different (McBride-Chang et al., 2013).

The co-occurrence we observed is also different from what has been found among English–Spanish bilingual children (55%) (Manis and Lindsey, 2010). This difference may be attributed to a higher similarity between English and Spanish. These two languages both have alphabetic writing systems, enabling deficits in one language to be easily transferred to the other. In contrast, as Chinese is a logographic language, cross-linguistic transfer between Chinese and English might be relatively weaker compared to that between Spanish and English. The hypothesis of low cross-linguistic transfer is supported by the small correlations found between Chinese and English reading-related cognitive skills (Yang et al., 2017). Additionally, another study (Pasquarella et al., 2015) examining the cross-language transfer of word reading in Spanish–English and Chinese–English bilinguals found that transfer of word reading accuracy is based on the structural similarities between the L1 and L2 scripts.

On the one hand, our finding of a 36% co-occurrence in reading difficulty across Chinese and English suggests that in addition to the assessment of L1 reading skill, the assessment of L2 reading skills is critical in early L2 readers, as a poor reader in one language may not necessarily be a poor reader in another language. On the other hand, this finding suggests that we need to pay additional close attention to L2 reading ability of poor Chinese (L1) readers, as reading difficulty in Chinese increased the possibility of being poor readers in English (L2).

Our second finding of sex differences in co-occurrence of reading difficulty is consistent with previous studies. The sex imbalance in dyslexia prevalence is well documented in alphabetic languages with a sex ratio ranging from approximately 3:1 to 5:1 in referred samples and from 1.5:1 to 3.3:1 in epidemiological samples (Shaywitz et al., 1990; Rutter et al., 2004). Recently, a meta-analysis including 16 studies (*N* = 552,729) concluded that males are more likely than females to be identified as having reading difficulties regardless of methodological and statistical influences (Quinn, 2018). Similarly, a within- and across-nation assessment of 10 years of Programme for International Student Assessment (PISA) data (Stoet and Geary, 2013) confirmed a male disadvantage in reading. The increased prevalence of dyslexia in boys versus girls has also been reported in China with a sex ratio from 1.6:1 to 2.0:1 in Cantonese speaking children in Hong Kong (Chan et al., 2007), and from 1.8:1 to 2.45:1 in Mandarin speaking children in Mainland China (Song and Wu, 2008; Zuo et al., 2010) in epidemiological samples. The sex gap could be due to sex differences in cognition (Kimura, 1999; Halpern, 2000), learning strategy (Poole, 2005; Griva et al., 2012), attitude toward second language learning (Davies, 2004), or a complex gene–environment interaction (Van Der Slik et al., 2015).

Our epidemiological data extend these previous studies by showing that the higher prevalence of reading difficulty in boys compared to girls was not only in L1, but also in L2. In fact, the sex difference was even more pronounced in L2 than in L1. Research on the impact of sex on L2 acquisition is much scarcer than on sex effects in L1 acquisition. Burstall (1975) reported that girls scored significantly higher than boys in learning French as a second language from age 13 to age 16 years. Davies (2004) further showed that this sex gap actually started as early as age 7 years, the first term when children started to learn French. The sex gap has also been reported in Korean learning English as a foreign language (Pae, 2004) and in adult learners of Dutch as a second language across countries of origin and continents (Van Der Slik et al., 2015). In terms of Chinese learners of English, Boyle (1987) reported that female Chinese students outperformed their male counterparts in English listening skills. To our knowledge, the current study is the first to report a significantly higher ratio of poor L2 reading in boys versus girls in Chinese children learning English as a second language.

Our third finding is the rural disadvantage in the prevalence of reading difficulty in L2 (English), with a larger disparity in L2 than in L1 reading. The urban–rural gap in reading performance has been frequently observed in large-scale studies related to L1 across countries such as PISA and Progress in International Reading Literacy Study (PIRLS) (Cartwright and Allen, 2002). These studies did not yield a consistent rural disadvantage, but revealed that rural–urban gap in reading performance in L1 varied in direction and magnitude across countries (Cartwright and Allen, 2002; Elijio and Urban, 2006). Most importantly, studies (Young, 1994; Cartwright and Allen, 2002; Elijio and Urban, 2006) have suggested that several factors contribute to the link between school location and reading performance. These factors include socio-economic environment (Elijio and Urban, 2006), school educational quality (Elijio and Urban, 2006), community differences in levels of adult education (Cartwright and Allen, 2002), and school–community connection (Tharp and Gallimore, 1991). One study (Wang et al., 2018) found that for Chinese primary students, the observed rural–urban reading literacy gap in L1 is mediated by parental education level and family literacy environment. In our study, the larger rural–urban gap in L2 compared to L1 reading abilities may reflect the fact that L2 reading is more susceptible to these above-mentioned factors. Moreover, children in lower grades are more vulnerable to L2 reading difficulty when compared to L1 reading difficulty, indicating that children’s L2 reading skill might be more influenced by the environment. One study (Kieffer, 2011) found that English L2 learners in the United States with initially limited English proficiency demonstrated English reading trajectories that were below national averages, but converge with peers from similar socioeconomic backgrounds after elementary school. Therefore, for children learning English as second language in China, those attending urban schools are more likely to have access to and benefit from more abundant L2 learning resources in family and school. Additionally, as children enter higher grades, they might receive more targeted tutoring in English education programs than do those who are in lower grades. In sum, the rural and lower grade disadvantage in the prevalence of reading difficulty in L2 observed in the current study could be attributed to one or more of these above factors, but will require more investigation to further our understanding.

Amplified by the Matthew Effect, the concept arising from findings that individuals who have advantageous early educational experiences are able to utilize new educational experiences more efficiently (Walberg and Tsai, 1983; Stanovich, 1986), the widening rural–urban gap is concerning. Poor readers in urban areas are more likely to be noticed, assessed, and to receive intervention with the help from well-educated parents, qualified teachers, and well-resourced urban settings. Studies have shown the significant education inequality of urban–rural area in China (Zhang et al., 2015), and rural children might be less likely to receive targeted instruction from the rural educational systems. These factors may lead to imbalances in the developmental trajectory of reading abilities between urban and rural areas, thus enlarging the disparity.

### Limitations and Future Directions

A number of caveats need to be noted regarding the present study. First, we used a lower end cutoff score of 25% to define reading difficulty; however, this arbitrary cutoff score approach has been critiqued for lack of stability over time (Francis et al., 2005). Nevertheless, the cutoff score approach remains one of the most common ways to define reading difficulties (e.g., Manis and Lindsey, 2010). Moreover, previous studies (McBride-Chang et al., 2013; Tong et al., 2015) investigating the prevalence of poor English reading in native Chinese speaking children also adopted this approach. Second, we aimed to recruit a representative sample of Beijing primary school children. For example, based on recommendations of local education administration officers, we recruited children from urban and rural schools with different levels of teaching quality and educational environment. Despite these efforts, the representativeness of our sample is open to discussion as we did not apply a completely random sampling approach. Finally, we admit that an English reading test would be the ideal instrument in screening English poor readers, but we only implemented a word spelling test. The reasons are that in China, English is mostly learned as a second language and we cannot directly use the standardized English reading tests developed for native English populations. Considering the practical issues and the time constraints imposed by the participating elementary schools, we used an English spelling test, which can be administered in large-scale settings and also can provide reliable and valid data regarding children’s current English reading abilities. Nevertheless, a richer set of reading tests as well as reading-related cognitive skill tests are needed in future research. Other psycholinguistic factors, for example, the age of acquisition should also be investigated to better depict the reading and cognitive profiles of reading difficulties in different writing systems such as Chinese and English (Davies et al., 2017). Employing multiple measurements as a means of identifying learners and assessing progress or future needs is recommended in order to develop a complete profile of a bilingual’s L1 and L2 language reading challenges.

## Conclusion

The overarching conclusion of the present study is that in Chinese–English bilingual children, despite striking differences between alphabetic and logographic writing systems, L1 reading difficulty still significantly increases the risk of L2 reading difficulty. This supports theories arguing for shared linguistic components in reading different writing systems, and underlines the importance of understanding the universality of reading between different writing systems. Furthermore, the male disadvantage and the urban–rural gap in the prevalence of reading difficulty call for special attention from the educational system and policy makers. These conclusions are only preliminary, and the need for more rigorous research of disadvantaged groups is evident.

## Data Availability Statement

The datasets generated for this study are available on request to the corresponding author.

## Ethics Statement

The studies involving human participants were reviewed and approved by the Beijing Normal University Ethics Committee. Written informed consent to participate in this study was provided by the participants’ legal guardian/next of kin.

## Author Contributions

All authors listed have made a substantial, direct and intellectual contribution to the work, and approved it for publication.

## Conflict of Interest

The authors declare that the research was conducted in the absence of any commercial or financial relationships that could be construed as a potential conflict of interest.

## Footnotes

1 http://cdi.cnki.net/
